# A Data Model for Using OpenStreetMap to Integrate Indoor and Outdoor Route Planning

**DOI:** 10.3390/s18072100

**Published:** 2018-06-30

**Authors:** Zhiyong Wang, Lei Niu

**Affiliations:** 1 GIScience Research Group, Institute of Geography, Heidelberg University, Im Neuenheimer Feld 368, 69120 Heidelberg, Germany; zhiyong.wang@uni-heidelberg.de; 2 School of Surveying and Urban Spatial Information, Henan University of Urban Construction, Pingdingshan 467036, China

**Keywords:** route planning, indoor, outdoor, OpenStreetMap

## Abstract

With a rapidly-growing volume of volunteered geographic information (VGI), there is an increasing trend towards using VGI to provide location-based services. In this study, we investigate using OpenStreetMap data to integrate indoor and outdoor route planning for pedestrians. To support indoor and outdoor route planning, in this paper, we focus on the connections inside buildings and propose a data model, which uses OSM primitives (nodes, ways and relations) and tags to capture horizontal and vertical indoor components, as well as the connection between indoor and outdoor environments. A set of new approaches is developed to support indoor modeling and mapping. Based on the proposed data model, we present a workflow that enables automatic generation of a routing graph and provide an algorithm to calculate integrated indoor-outdoor routes. We applied our data model to a set of test cases. The application results demonstrate the capability of our data model in modeling built environments and its feasibility for the integration of indoor and outdoor navigation.

## 1. Introduction

With the expansions of urban population and built environments, the number of human activities in both indoor and outdoor spaces is also increased, which creates a growing need for spatial information to support a wide range of applications, such as route guidance, emergency management, cadaster and land management [1,2].

One of the important applications of spatial information is to support integration of indoor and outdoor navigation. As outdoor navigation has been widely investigated and many approaches have been proposed for indoor modeling and routing [3,4,5], increasing attention has been devoted to the integration of indoor and outdoor navigation. The work in [6] examined the widely-used route planners (such as Google map, OpenRouteService, Bing) and showed that these planners provide limited indoor/outdoor routing functionality. By combining the ground transportation network with the building network, [7] developed a 3D GIS-based emergency response system that can provide real-time navigation guidance for responders to research rescue points inside buildings. The work in [8] proposed a geometric network model, which extracts building information from IFC (Industry Foundation Classes) and uses the entrance-to-street strategy to integrate indoor and outdoor networks for route planning. To support path finding on a campus, [9] proposed an XML-based approach to construct a topological model of built environments from floor plan data, defining a set of portal types for outdoor spaces, as well as building features (e.g., stairs, doors and elevators). The work in [10] used an object-based approach to model connectivity inside buildings and outdoor networks, considering the multiple modes, such as bus and pedestrian. However, the data sources used by the above systems have a huge diversity in data structure, data coverage, data update and data availability, which makes it very difficult to apply these systems to other places.

In the past two decades, volunteered geographic information (VGI) has gained great interest in the geoscience communities [11,12]. VGI relies on citizens as sensors [13] and represents a new data collection method that uses humans to gather data about the Earth and its environments. One of the most successful VGI projects is OpenStreetMap (OSM), which is a worldwide map created by volunteers. OSM uses three basic data types (i.e., nodes, ways and relations) and tags (key-value pairs) to describe real-world objects. It has a central database that allows users across the world to access, edit and download geographical data. Through years of efforts of interested volunteers, the OSM database has stored a huge spatial dataset of the environments with very rich metadata, covering major cities in the world. Although there are some concerns about OSM data quality and data reliability [14,15,16], OSM has been been studied by many works and used in a wide range of applications, such as location-based services [17], disaster management [18] and land administration [19]. Especially in the field of navigation, the OSM database contains a vast amount of transportation features (e.g., roads, bus stops, sidewalks) that have been used for outdoor navigation and provides a very promising solution for collecting and maintaining the data for indoor navigation [20].

With the rapid development of indoor positioning and sensor techniques, such as simultaneous localization and mapping (SLAM), WIFI fingerprinting, Bluetooth and inertial measurement units (IMU), many efforts have been devoted to indoor mapping in the academic field [21,22,23] and in industry (e.g., indoor Google Maps, Bing Venue Maps, NAVTEQ Destination Map). In the past years, there has also been an increased interest in indoor mapping within the VGI community to use OSM basic elements to capture indoor objects and their relationships in built environments [20,24]. Some indoor mapping projects have been carried out to map certain types of built environments (e.g., universities, train stations), which lead to a couple of indoor maps, e.g., OpenLevelUp (www.openlevelup.net), OpenStationMap (www.openstationmap.org) and OSMTools indoor map (http://clement-lagrange.github.io/osmtools-indoor). Moreover, there is also some research that has studied the use of the OpenStreetMap framework to collect indoor data of the studied areas and to use them to provide location-based services for intended users, such as university students and people with disabilities [25,26]. The above-mentioned works provide a rich set of data, tools and models, which give useful insights into the further developments of OSM-based approaches for indoor mapping.

For the collection and management of building information, a data model is needed to describe indoor spaces for navigation applications. Typical data models for modeling indoor environments are the building information model (BIM) and the City Geographic Markup Language (CityGML). A representative example of BIM is the Industry Foundation Classes (IFC). CityGML is an Open Geospatial Consortium (OGC) standard. Both BIM and CityGML consist of a number of classes and contain highly detailed and semantic information on the built environments. Based on these models, some researchers have developed a variety of methods to generate graphs for navigation purpose [3,8]. However, these models carry too much architectural information such as structural and reinforcing information, which are mainly designed for visualization purposes and not essential for navigation. Along with the indoor mapping activities of the VGI communities, a set of indoor modeling and mapping proposals for OpenStreetMap has emerged. Based on 3D building modeling frameworks and ontologies, [20] proposed IndoorOSM with the intention of mapping all relevant information for indoor routing. Although it has some deficiencies (e.g., directly using OSM IDs to describe the connections between floors) and is currently not embraced by the OSM community, some of its proposed tagging schemes can still be used for the mapping of indoor objects like rooms, windows and doors. Simple Indoor Tagging [27] is also a tagging schema proposed for indoor mapping and mainly focuses on public buildings (e.g., train stations). Another indoor-related proposal is the Full 3D Buildings (F3DB) schema [28], which is designed with concern for its compatibility with existing standards, such as CityGML and IFC. However, these tagging schemas lack consideration of detailed mapping of the connections between indoor elements (e.g., stairs), which would cause problems in deriving the actual geometric distance for indoor routing.

In this study, we focus on pedestrian routing and aim to provide seamless integrated indoor-outdoor navigation service, using OpenStreetMap data. To support indoor mapping and routing, we design and develop a new data model based on the OSM basic data structure, focusing on mapping the connections in built environments. A set of novel approaches is proposed to model building components to structure their geometric and topological information in an effective and consistent way. Based on the classical Dijkstra algorithm, we propose a new routing algorithm to generate indoor-outdoor routes. The remainder of this paper is organized as follows: In Section 2, we describe our OSM-based data model in detail. In Section 3, we present a workflow of constructing a routing graph based on the proposed data model and describe the algorithm for the integrated indoor-outdoor route planning. In Section 4, we apply our data model and the algorithm to a set of scenarios and show the application results. In Section 5, we give our conclusions and discussions and end this paper with some suggestions for future research.

## 2. The Proposed Data Model

To use OSM data for integration of indoor and outdoor navigation, a data model is required. Such a model will not only be used to guide mapping of indoor environments, but also support storage, processing and analysis of the data. Based on the experience gained with previous works, we derive the following requirements that the data model should fulfill: (1) It should be as simple as possible. This is because the OSM community is composed of professional members, as well as normal citizens who do not have professional skills or GIS knowledge. (2) It should be able to describe all relevant (geometry and topology) information that is essential for the integration of indoor and outdoor routing. (3) It should be consistent with the OSM basic structure and be as compatible as possible with other existing data tagging schemas.

**Figure 1:**
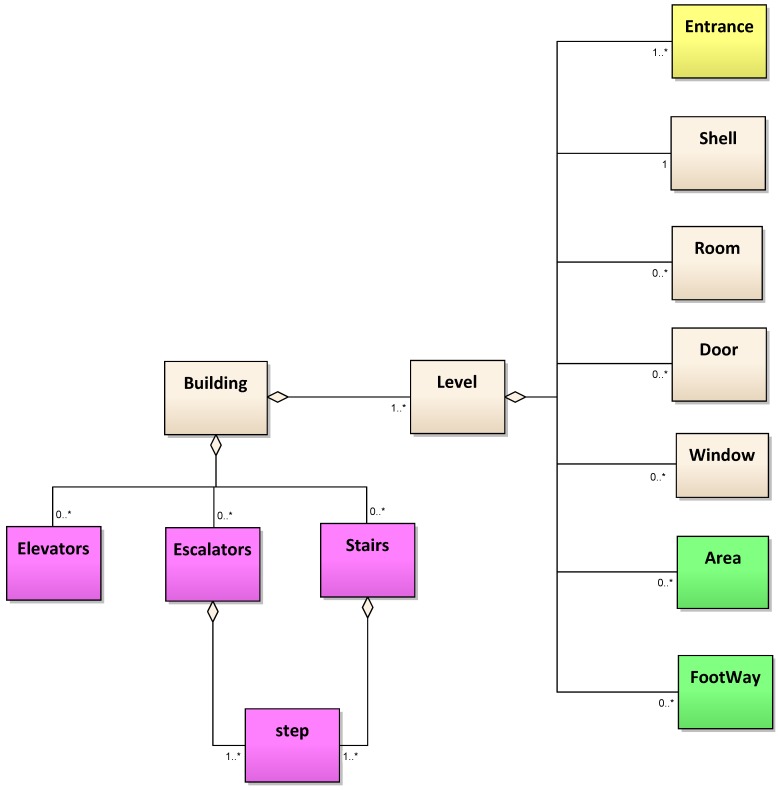
The UML diagram of the proposed data model.

Following the above requirements, we design a data model, modifying the indoor tagging schema from the previous work of [20], and focus on modeling the connections between building parts. A set of new approaches is proposed to map the connecting spaces of inter-/intra-floors inside buildings. Based on building footprint data, we adopt OSM nodes and ways to represent the location and shape of indoor components, using semantics to capture the third dimension. Besides, OSM relations are also used to describe the connections between various indoor parts. Our approaches allow the model not only to store outer characteristics and the inner building components, but also to capture the connectivities between them, which enables the use of 3D information to generate routes that are close to the real environments. Figure 1 shows the UML diagram of our data model. Basically, the modeling of built environments in the proposed model consists of four parts: (1) modeling of floors and inner building parts (in light gray); (2) modeling of horizontal elements inside floors (colored in green); (3) modeling of vertical elements that connect floors (colored in purple); (4) modeling of connections between outdoor and indoor environments (colored in yellow). In the following sections, we will describe the proposed data model in more detail.

### 2.1. Modeling of Buildings and Inner Building Parts

**Figure 2:**
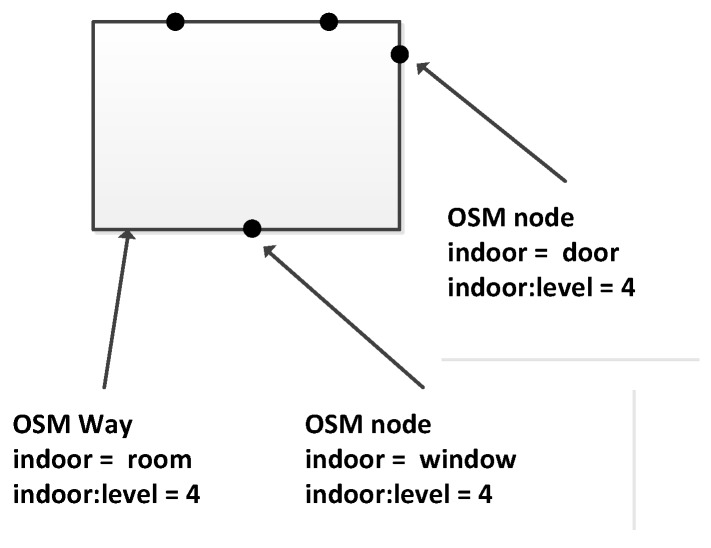
Modeling of a room with windows and doors.

Following the approach proposed by IndoorOSM, we use an OSM relation to represent a building, which has general building information attached, such as address, postcode and name. Each building relation is a main relation and composed of level relations (representing floors). Each level contains its outer boundary (shell), level number and height, which is the distance between the current level and the next level above. For each level, we model inner building parts in a similar way to IndoorOSM, but an additional tag “indoor:level = *” (* is a wildcard standing for any tag value) is introduced for each building part to clearly indicate the level to which this building part belongs. This would help facilitate the editing and processing of the OSM indoor data. Each room is defined by a polygon and can have some openings, such as windows and doors. The location information of the openings (doors and windows) is provided with a node representing their centric position, as shown in Figure 2. Note that although in normal situations, windows are not used for navigation purposes, they are very important for routing in some special situations (e.g., fire evacuation).

### 2.2. Modeling of Horizontal Connections

**Figure 3:**
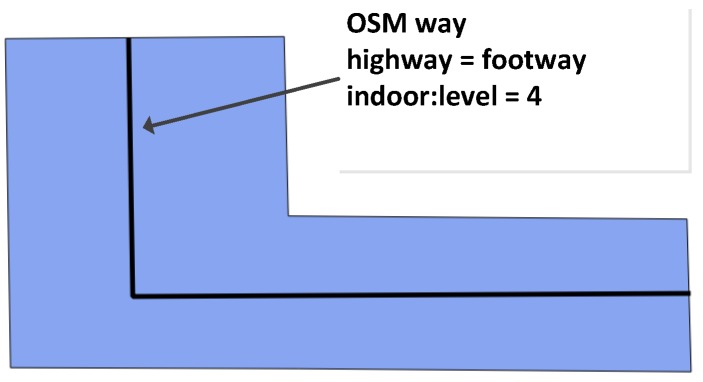
An area with a regular shape.

**Figure 4:**
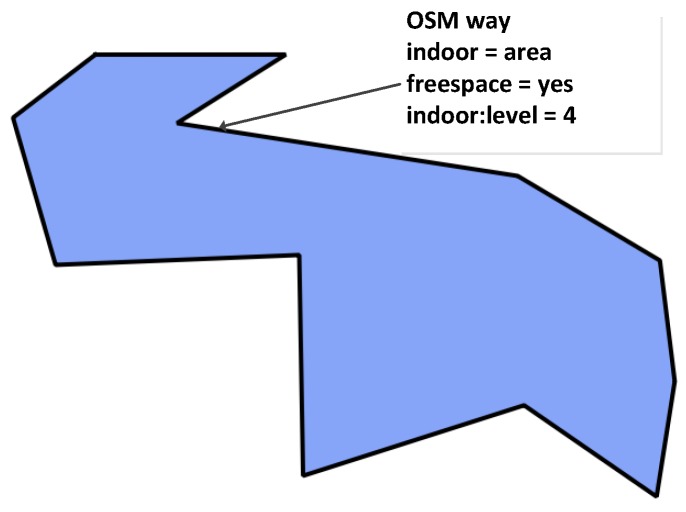
An area with a complex shape.

Indoor horizontal spaces, such as corridors, halls and passages, play an important role in the navigation at each floor of a building, connecting rooms and doors. These spaces are usually areas that are without walls and are where people can walk freely. In the new model, we use an OSM closed way to map the outline of each indoor space and attach the tags “indoor = area” and “indoor:level = *”. Because these indoor areas can have not only regular shapes (see Figure 3), but also complicated geometries (see Figure 4), in the new data model, we apply two different ways to represent the horizontal connections at each floor: (1) For regular shapes (like Figure 3), we use a direct mapping approach and explicitly map foot ways that people can walk along inside buildings. Each indoor foot way is represented by an OSM way, attaching the tags “highway = footway” and “indoor:level = *”. The indoor foot way can be directly derived from floor plans. Such an approach allows foot way information to be directly integrated into the generation of navigation graphs, without additional processing of the geometric information of indoor spaces. (2) For the areas with complex geometries (like Figure 4), we introduce a new tag “free_space = yes” and attach it to the way of the indoor space. This tag indicates that special processing is needed to derive an appropriate pedestrian-friendly network for routing. More details about deriving navigation graph from complicated geometries will be presented in Section 3.1.

### 2.3. Modeling of Vertical Connections

**Figure 5:**
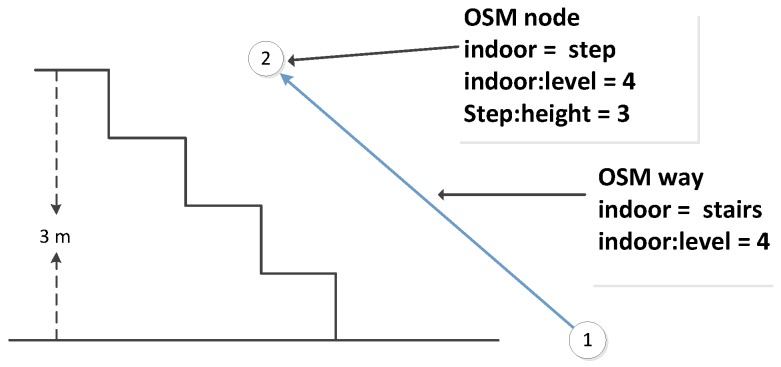
An example using OSM ways to model stairs.

**Figure 6:**
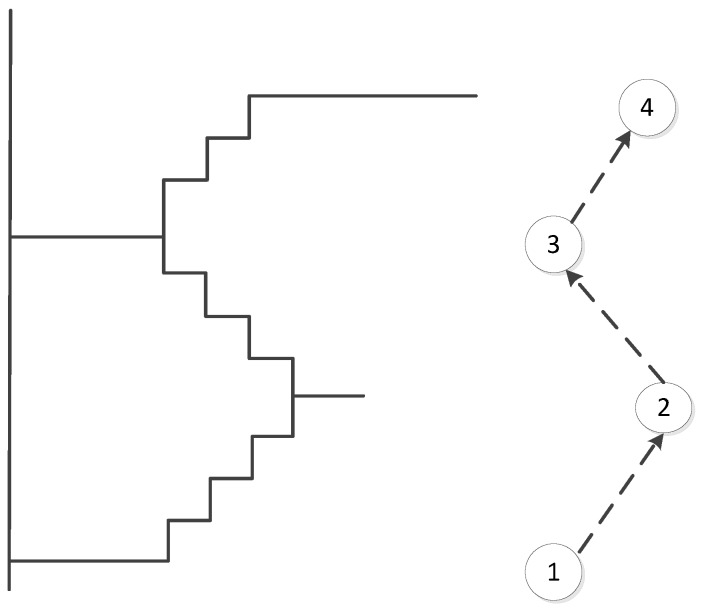
An example of using OSM relations to model stairs. The stair relation here consists of four members (i.e., Step 1, Step 2, Step 3, Step 4).

**Figure 7:**
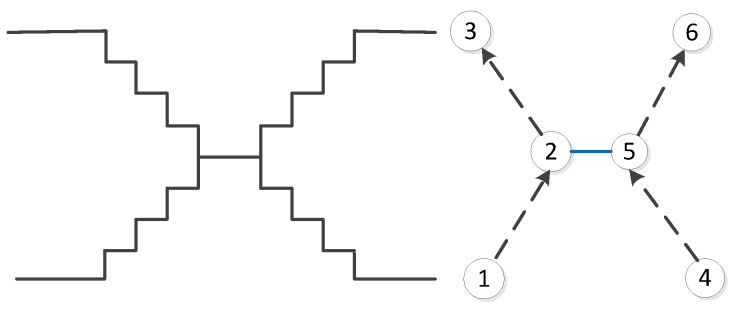
An example of using OSM ways and OSM relations to model stairs. The stairs here are split into three parts: (1) a relation with members Step 1, Step 2 and Step 3; (2) a relation with members Step 4, Step 5 and Step 6; (3) an OSM way representing a part of stairs (in blue).

**Figure 8:**
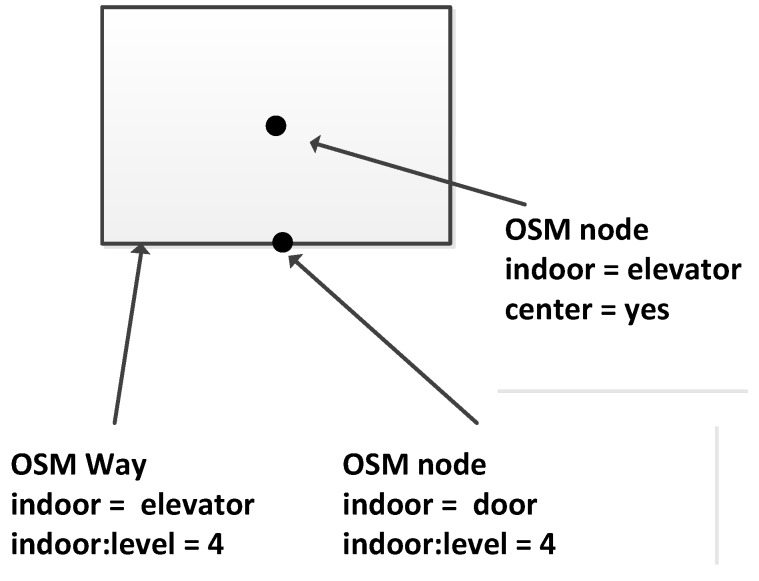
An example of using an OSM way and node to model an elevator.

Stairs, escalators and elevators are typical vertical elements that connect different floors. Regarding the stairs, in our data model, we propose three ways to model them: (1) We use the same method that is used for outdoor stairs and explicitly map the indoor stairs as OSM ways; see an example in Figure 5. Because a staircase may consist of several steps, we select a subset of steps and map only transit steps (steps on floors or landings). These steps are modeled as OSM nodes, and contain “indoor:level = *” and “step:height = *”, which denotes the height relative to its level. Such information will be used to derive the actual height of the step that is above the ground. (2) For some cases that are difficult to handle for direct mapping (e.g., the connecting points of stairs are quite close to or overlapping with each other), we introduce OSM relations to represent the stairs and add step nodes into stair relations as members, as shown in Figure 6. It should be noted that the step nodes in the stair relation are ordered from lowest step (starting node) to the highest (ending node). (3) Considering some staircases may have a complex topology between floors, we propose to partition them into a limited number of parts based on certain rules (e.g., using visibility criteria). So that we can combine Methods (1) and (2) mentioned above, and use a way or a relation to map each part individually (see Figure 7). With respect to escalators, they are modeled in a similar way as stairs, but they have additional tags attached, e.g., “conveying = yes”, “incline = up” and “incline = down”, to indicate the moving direction of the escalators. For elevators, we map each elevator as a relation, which is comprised of closed ways. Each way represents the boundary of an elevator shaft on each floor. It should be mentioned that the elevator way should be combined with a door node, which is linked with the indoor networks on the same floor, and with a center node, which connects the networks of different floors. An example is shown in Figure 8.

### 2.4. Modeling of Connections between Indoor and Outdoor Environments

**Figure 9:**
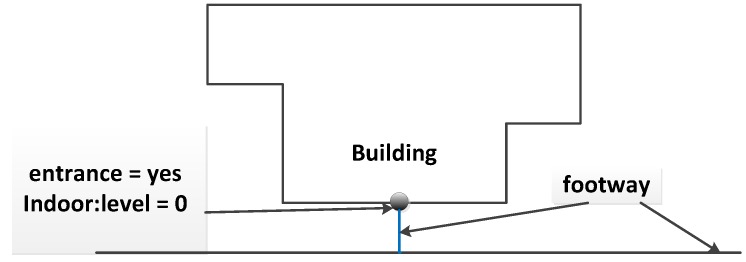
An example of using OSM ways to model the connection between indoor and outdoor environments. An OSM way (in blue) is added to connect an entrance to an outdoor network.

**Figure 10:**
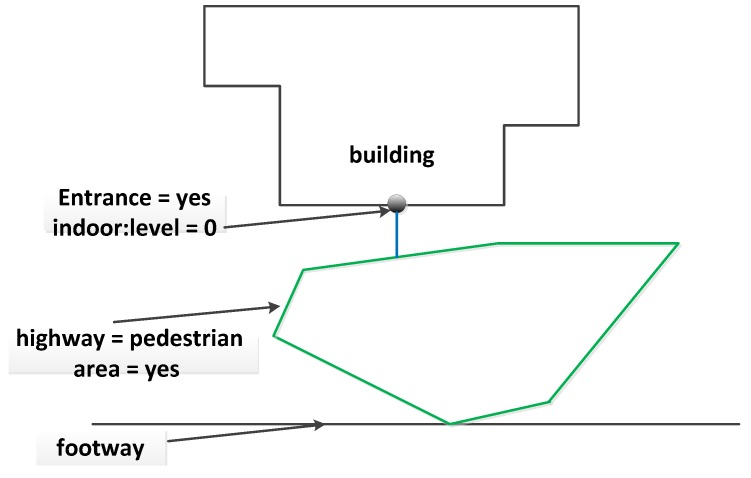
An example of using OSM closed ways to model the connection between indoor and outdoor environments. A way (in blue) and an area (in green) are created to link indoor and outdoor networks.

Entrances and exits are the main building components used by people to enter or exit the buildings and play an important role in connecting indoor and outdoor networks. In this study, the entrances and exits of buildings are modeled as OSM nodes with tags “entrance = yes/exit”, and “indoor:level = *”. They are connected to indoor networks of the same floor. To integrate the indoor networks with street networks, in this paper, we apply two mapping approaches: (1) explicitly mapping the path to entrances/exits; an OSM way is added to represent the foot ways that link outdoor road networks and entrance/exit nodes, as shown in Figure 9; (2) using OSM closed ways and mapping the open areas that connect indoor and outdoor environments; the closed ways have the tag “area = yes” attached and are connected to entrances/exits and outdoor road networks (see Figure 10).

## 3. Indoor-Outdoor Route Planning

Base on the data model described in Section 2, in this section, we present a workflow for generating a 3D routing graph and provide a routing algorithm, which uses the building information to find the optimal paths.

### 3.1. Workflow for Generating the Routing Graph

**Figure 11:**
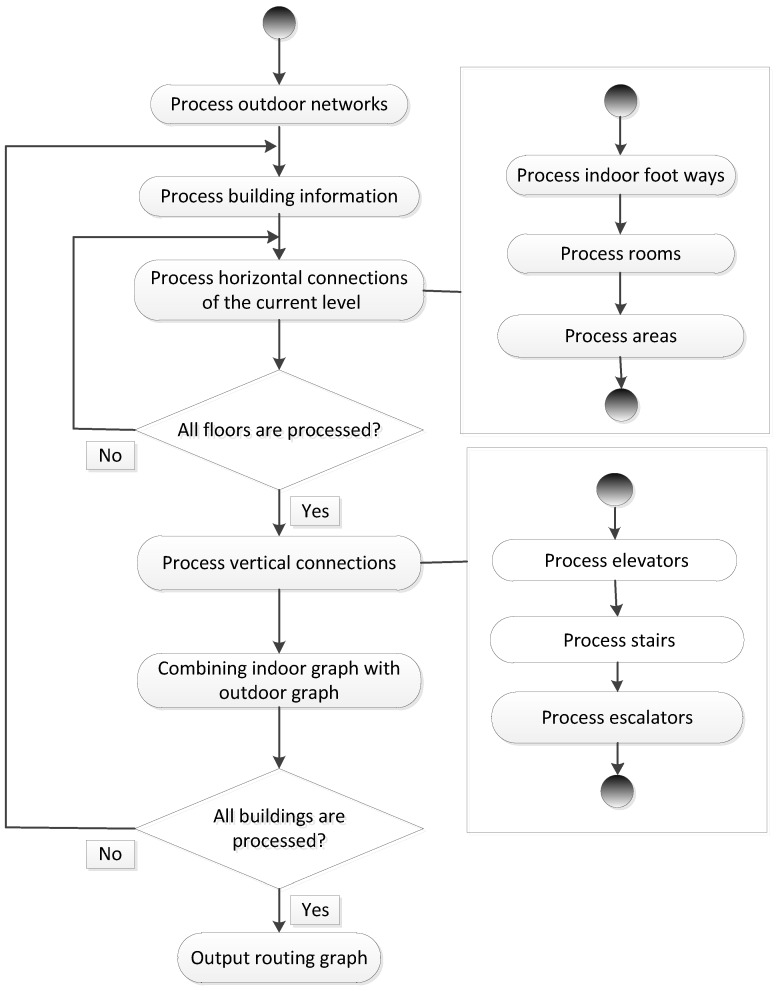
The workflow of generating the routing graph.

To combine indoor and outdoor route planning, a 3D routing graph is required. In this section, we provide a workflow that gives a guideline for processing the data of outdoor networks and the building components of different types. Basically, our workflow consists of five steps (as shown in Figure 11):

1. Processing outdoor networks: We first process the OSM ways that can be used by pedestrians in outdoor environments and include them in the routing graph.
2. Processing building information: For each building relation, the height information of all floors is collected to derive the elevation of each floor. For the OSM nodes of each floor, we derive their height based on the height of the associated floor and their distance relative to the floor. This would allow the use of 3D information to obtain the actual length of edges in the routing graph.
3. Processing horizontal connections: Within each floor, we directly add indoor foot ways to the indoor navigation graph. For each room, a centroid node is generated based on its boundary and is connected to the doors of the room. For indoor open spaces, we first check that they contain the tag “free_space = yes”. If they do, we create a new sub-graph with a set of new edges using the visibility graph approach [29] and merge it with the indoor network.
4. Processing vertical connection: Stairs and escalators are processed in a similar way. We extract the OSM nodes that represent stairs and escalators along with their sequence and create a new edge between each pair of adjacent OSM nodes. Once this is done, the direction constraint (up or down) is obtained from the related tags of OSM ways and stored as an attribute of edges. For each elevator and each floor, we generate a center point based on the location of the elevator center and link it to the corresponding door of the elevator. The center points of the elevator at different floors are also connected to their adjacent points.
5. Combining indoor networks with outdoor networks: The routing graph generated from one building is merged with the whole routing graph.

The above steps from (2)–(5) are executed accordingly for each building. After all buildings are processed, a complete 3D routing graph is constructed and will be used for route planning.

### 3.2. Routing Algorithm

To calculate indoor-outdoor routes, we design and develop a routing algorithm based on the Dijkstra algorithm [30] and try to minimize the travel time of paths. Because the size of the routing graph is largely increased by combining indoor and outdoor networks, searching the entire graph to find the optimal results would be very time consuming. To reduce the computational overheard, in the algorithm, we provide an approach that uses the entrance information of buildings to prune the edges and nodes of the irrelevant buildings. For each indoor edge, we also store the type information of its corresponding building component and derive the time needed for traveling along the edge accordingly.

**Figure 12:**
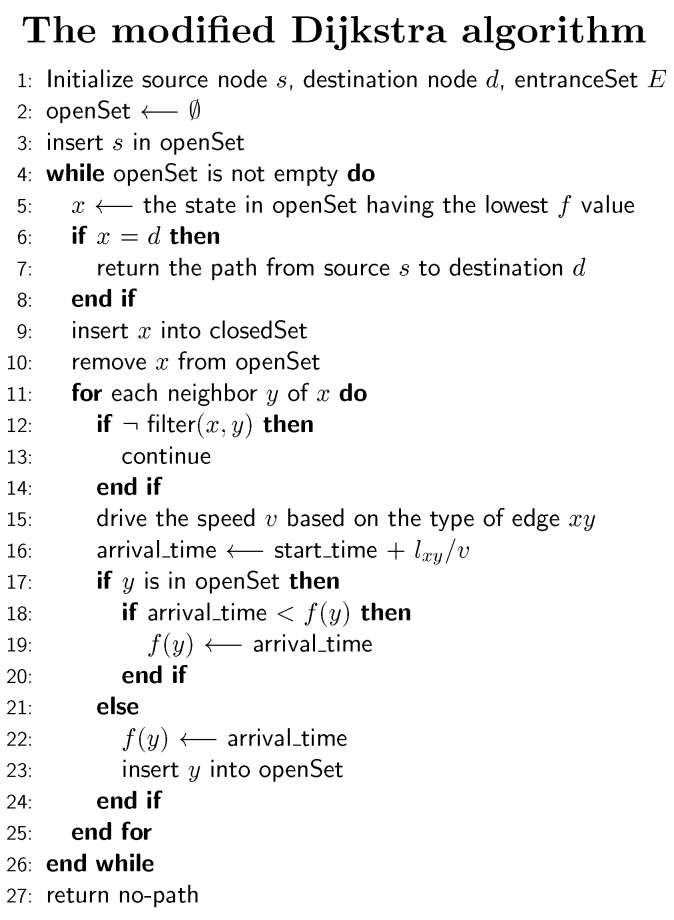
The modified Dijkstra algorithm for indoor-outdoor route planning.

**Figure 13:**
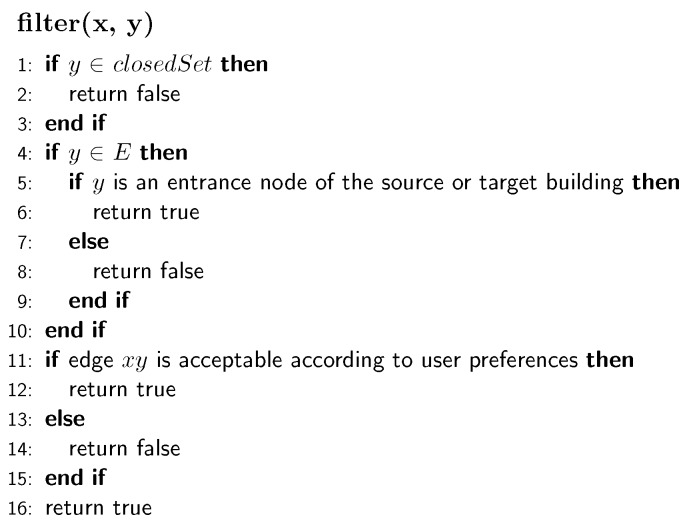
The function for filtering edges and nodes.

Figure 12 shows the structure of the modified algorithm for indoor-outdoor route planning. In the initialization of the algorithm, the developed algorithm is given with two nodes, i.e., the source node and target node. Each of these two nodes corresponds to either a room in a building or a point in outdoor road networks. Following the basic principle of the Dijkstra algorithm, the algorithm maintains two sets: openSet, which stores the nodes waiting for extensions, and closedSet, which stores the nodes that have been extended. In our algorithm, we introduce a new set entranceSet to store the entrance nodes of all buildings and use it for filtering nodes. The algorithm first selects the node *x* with the lowest cost, expands it to generate its successors and uses them to update the openSet. In the generation of successors, we introduce a filter function for filtering out irrelevant edges and nodes (Figure 13), limiting the searching space. The filter performs three functions: (1) skip the next node if it is in closedSet; (2) check if the next node is an entrance node and which building it belongs to and remove it from extensions if it does not belong to the target building or the source building; (3) check if an edge should be filtered out according to user preferences (e.g., using elevators or stairs). In Line 15 (Figure 12), we derive the traversing speed of edges based on the types of the corresponding building parts: (1) for elevators and escalators, the speed is directly extracted from OSM data (e.g., elevator speed = 5 m/s, escalator speed = 2 m/s); (2) regarding stairs, we calculate the gradient of the stairs and use it to obtain the ascending and descending speeds, according to the model provided in the works of [31]. From Line 17–24 (Figure 12), the successor *y* with the newly estimated arrival time is examined and is used to update openSet for further extensions.

## 4. Application Results

Using the data model presented in Section 2, we have mapped two buildings of the campus of Heidelberg University: the Mathematikon building (Berliner Str. 45) and the Geography building (Im Neuenheimer Feld 348). The Mathematikon building contains seven floors (five above the ground and two floors underground), which are connected by a couple of stairs and elevators. On each floor, there is a group of rooms with different usages. In the Geography building, it consists of two floors and one stair. We gathered the indoor data based on the evacuation plans of the buildings, using the Java OpenStreetMap Editor (JOSM). A Java-based routing engine has been built, employing the modified Dijkstra algorithm (presented in Section 3.2) to calculate the shortest route. Based on a JavaScript library named osmbuildings (www.osmbuilidng), we developed a web application tool to render geo-data on 3D maps [32] and used it for 3D visualization of the indoor environments and the computed routes in this study. Our integrated indoor and outdoor route planning system was tested in three cases: (1) navigation within one building; (2) navigation from an outdoor point to a room inside a building; and (3) navigation between two rooms in different buildings. The results from the application to these two cases are presented in Section 4.1, Section 4.2 and Section 4.3, respectively (see https://www.youtube.com/watch?v=60Ab7f8YZ7w for a demonstration).

### 4.1. Case 1: Navigation within One Building

**Figure 14:**
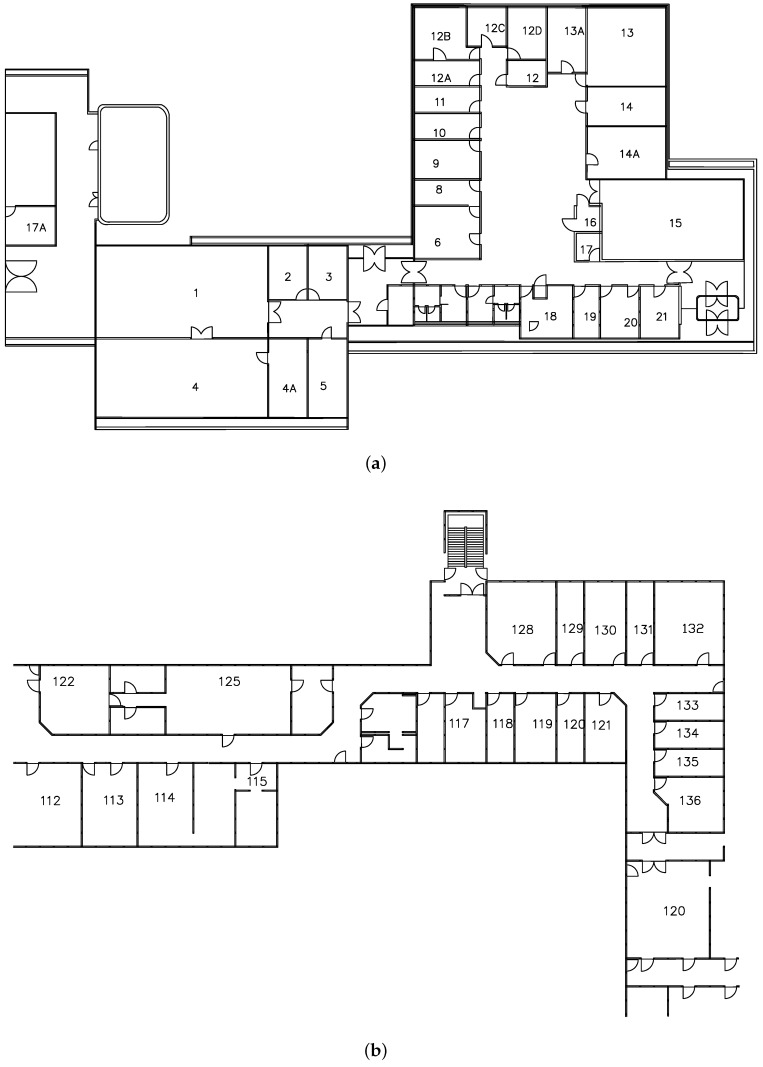
The floor plans of the Geography building. (**a**) Floor plan of the ground floor; (**b**) Floor plan of the first floor.

**Figure 15:**
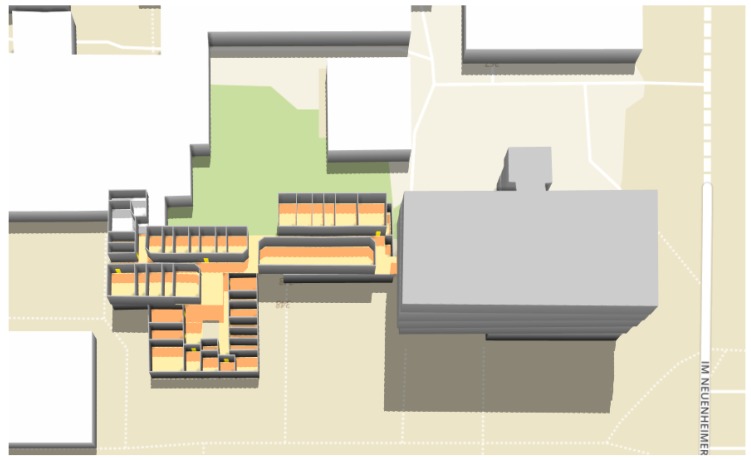
3D visualization of the Geography building.

**Figure 16:**
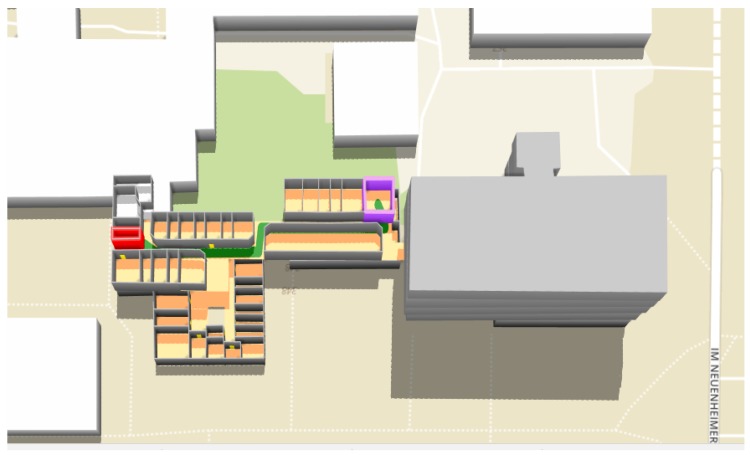
Route R8 from Room 133 (in red) to Room 112 (in purple) in the Geography building.

In order to test the effectiveness of our proposed data model and algorithm for indoor routing, we select the Geography building as the testing environment and apply our system to generate indoor routes. Figure 14 shows the floor plans of the building. Figure 15 shows the 3D representation of the building constructed from our indoor OSM data model. We randomly selected 10 pairs of rooms, which are located on the two floors. For each pair of rooms, we used our system to generate indoor routes between them and compare the results with the actual distances as a reference. Table 1 shows the calculated route results. An example of the generated indoor routes (i.e., R8) is shown in Figure 16. The actual distances are measured in the building and are obtained by using a smartphone pedometer application (for measuring horizontal distance) and a tape measure (for measuring vertical distance). We calculated three standard errors: the mean absolute error (MAE), the mean squared error (MSR) and the mean absolute percentage error (MAPE). In this case, the MAE, the MSR and the MAPE are 1.11 m, 1.87 m and 3.22%, respectively. As you can see, the MAPE is below the standard threshold for errors (5%), which demonstrates the feasibility of our approach in indoor modeling and routing.

### 4.2. Case 2: Navigation from an Outdoor Point to an Indoor Room

**Figure 17:**
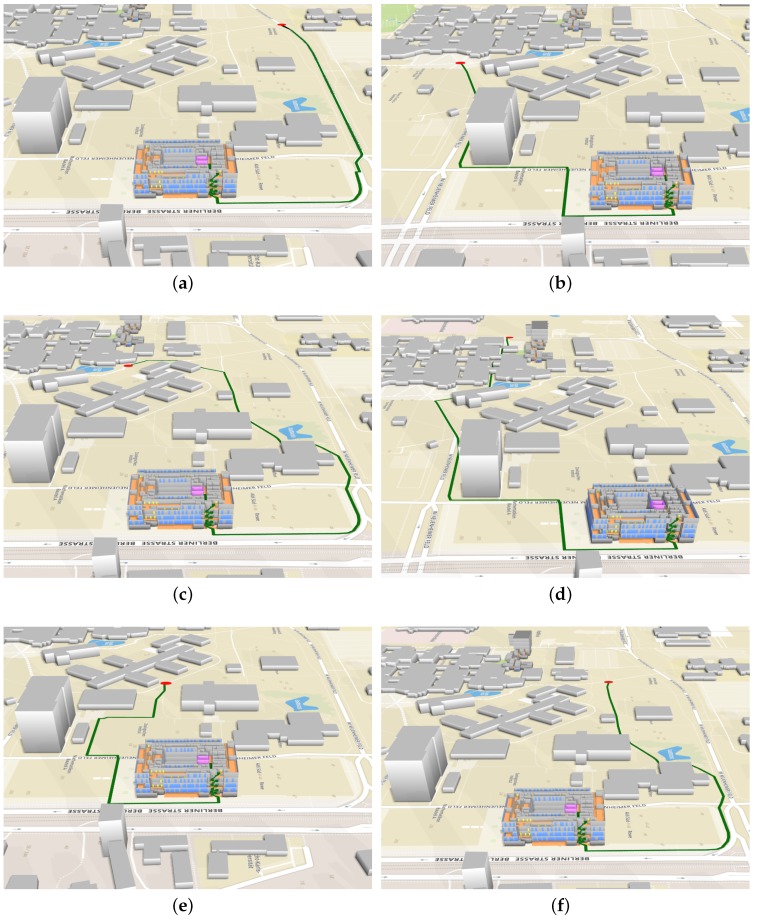
An overview of integrated indoor and outdoor routes (in green) from three outdoor points (in red) to a room (in purple) in the Mathematikon building. The windows and the doors of the buildings are colored in blue and in yellow, respectively. (**a**) Route R1; (**b**) Route R3 (**c**) Route R5; (**d**) Route R7; (**e**) Route R9; (**f**) Route R11.

In this section, we present several navigation scenarios in which people have to be routed from different outdoor points to a room on the fourth floor in the Mathematikon building. We apply the system to perform integrated indoor and outdoor routing and compare the routing results obtained by the classic Dijkstra algorithm (given the average walking speed of 5 km/h) and by our modified Dijkstra algorithm. Figure 17 shows the visualization of the calculated integrated indoor-outdoor routes for the considered scenarios. As we can see from the figure, our route planner can not only give the routes to the target building, but also provide indoor paths that guide the users to the target room. Table 2 shows more details of the integrated routes. Note that routes R1, R3, R5, R7, R9 and R11 are calculated by our modified Dijkstra algorithm, and R2, R4, R6, R8, R10 and R12 are calculated by the classic Dijkstra algorithm. As shown in the table, our modified algorithm finds the same shortest paths as the classic Dijkstra algorithm does, but gives a different estimation of traveling times, considering the speed reduction during climbing stairs. The results also show that by using the entrance information of buildings, the proposed algorithm can skip the nodes that are inside irrelevant buildings, which reduces the search scope and increases the efficiency of finding optimal paths in urban environments.

### 4.3. Case 3: Navigation between Two Buildings

**Figure 18:**
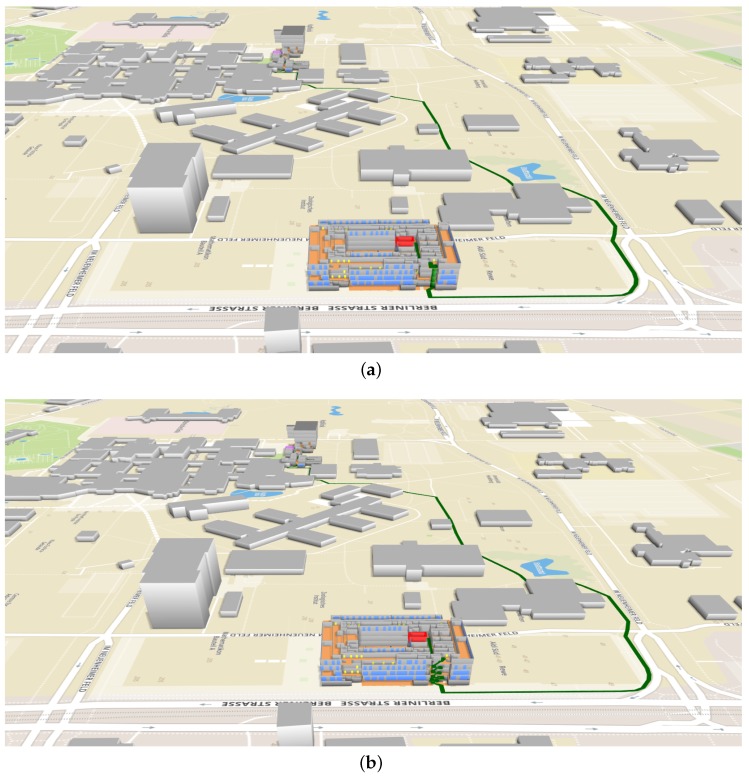
Examples of the calculated routes (in green) from the source room (in red) in the Mathematikon building to the target room (in purple) in the Geography building. The windows and the doors of the buildings are colored in blue and in yellow, respectively. (**a**) R7 (using elevators); (**b**) R8 (without using elevators).

This section shows the case that users have to navigate between the two different buildings. Here, we assume that users have to travel between a room in the Geography building and a room in the Mathematikon building. Five pairs of rooms were randomly selected, one from the Geography building and the other from the Mathematikon building. For each pair of rooms, the navigation system determines the fastest route in the 3D routing graph, according to their preferences (using elevators or not). Figure 18 shows an example of the planned routes (in green) that connects the source building and the target building. As you can see, the system generated two different routes based on users’ preferences. Table 3 shows the calculated route results. Note that R1 and R2, R3 and R4, R5 and R6, R7 and R8, and R9 and R10 have the same source and target rooms, respectively. However, they have different travel times, which are derived based on the descending and ascending speeds of moving through stairs and elevators along the routes. As one can see from the table, users who prefer using elevators have shorter routes and spend a lesser amount of time to arrive at the target rooms than the users who prefer using stairs. Through the 3D visualization, users can not only easily identify which part of the building the target room is located in, but also have a good overview of indoor and outdoor paths, especially the building parts connecting different floors.

## 5. Conclusions and Future Works

Integration of indoor-outdoor navigation services is useful in a variety of applications, ranging from daily activities to emergency response. In this study, we focus on using OpenStreetMap data to combine indoor and outdoor routing for pedestrians. By extending the previous works on IndoorOSM, in this paper, we have developed an OSM-based data model and a set of new approaches to describe the geometry and topology of indoor components, especially the connections between different floors of buildings. A workflow has been provided to give guidelines to automatically derive the routing graph combing indoor and outdoor networks. To make the routing more efficient in the combined network, we have extended the Dijkstra algorithm and use the entrance information of buildings to limit the search scope. We applied the proposed data model to map selected buildings and employed the extended algorithm to perform indoor-outdoor routing. The application results not only indicate the feasibility of the proposed data model in maintaining the 3D information and handling the complex topology of building structures, but also show the efficiency and effectiveness of the proposed algorithm in supporting indoor-outdoor route planning.

Although we have demonstrated the capability of the developed data model and routing algorithm for the integration of indoor and outdoor route planning, there are some aspects that have not been reflected in the current developments and that should be mentioned. First of all, there is not yet a route instruction engine available in the system to provide guidance for users. Commands such as “Go upstairs”, “Go downstairs”should be generated in the form of voice or text, according to the direction and location of users. The development of such an instruction engine will largely rely on the advancement of the indoor and outdoor positioning technology. Second, in the current data model, we only consider the information essential for routing; some building information, e.g., roof type, window geometries and thickness of walls, are not taken into account. The structuring of such information is outside of the scope of this paper. Third, our approach can not only provide navigation guidance to the destinations in the buildings above the ground, but also can support indoor routing under the ground. However, due to the limitations of the 3D map visualization technology, how to present the indoor routes and the environment underneath the ground surface is still an open issue and requires further investigation. Last, but not least, currently, the indoor data are collected manually by digitizing the floor plans, which could introduce various errors to the data. Because the reliability of indoor routing results largely relies on the accuracy of indoor data, new methods would be needed for the evaluation of the quality of indoor data.

In future works, we would like to investigate indoor routing for disabled people (e.g., wheelchair users). In indoor environments, some of the building components are not accessible for special users and should be considered obstacles in the route determination. For example, people in wheelchairs cannot use either the stairs or some doorways if they are not wide enough. To deal with these situations, the data model should be adapted to support mapping and storage of this information. A customized routing graph for disabled people would also be required. Furthermore, we will study the conversion between the BIM model (e.g., CityGML, IFC) and our data model. Such a conversion will not only enable the enrichment of the OSM database with existing 3D datasets, but also extend the use of OSM data to other application domains, such as energy modeling and urban planning. To make the mapping of indoor objects between our proposed model and BIM models possible, new transformation rules need to be built. Another important research direction is to integrate indoor and outdoor route planning for first responders in the presence of obstacles. During emergencies, indoor environments can be very dynamic due to the occurrence of moving obstacles (e.g., fires, plumes). These dynamic factors can affect the availabilities of indoor spaces and should be considered in the data model to provide routes avoiding obstacles. Last but not least, we plan to integrate other travel modes (such as car and bus) in the combined indoor and outdoor route planning. New routing algorithms would be needed to take into account different combinations of travel modes and to identify optimal transfer points between modes.

## Figures and Tables

**Table 1:** Calculated results for Case 1 (R, route).

Route ID	Starting Room	Ending Room	Calculated Distance (meters)	Reference Distance (meters)	Distance Difference (meters)	Relative Error
R1	21	12C	33.4	31.9	+1.5	4.7%
R2	12	3	40.0	39.9	+0.1	0.2%
R3	20	11	25.7	25.1	+0.6	2.6%
R4	2	19	34.7	32.5	+2.2	6.8%
R5	9	21	26.3	26.1	+0.2	0.8%
R6	6	12D	22.2	22.8	−0.6	2.6%
R7	117	20	38.5	40.3	−1.8	4.5%
R8	133	112	53.2	50.7	+2.5	4.9%
R9	112	117	21.9	22.8	−0.9	3.9%
R10	19	133	51.0	51.7	−0.7	1.4%

**Table 2:** Calculated results for Case 2.

Scenario	Route ID	Total Travel Distance (meters)	Total Travel Time (min)	Number of Visited Nodes
S1	R1 R2	647.3 647.3	8.1 7.7	790 911
S2	R3 R4	602.8 602.8	7.6 7.2	785 907
S3	R5 R6	730.9 730.9	9.1 8.8	880 1029
S4	R7 R8	925.4 925.4	11.5 11.1	981 1138
S5	R9 R10	482.7 482.7	6.2 5.8	650 757
S6	R11 R12	647.6 647.6	8.1 7.8	746 898

**Table 3:** Calculated results for Case 3.

Pair No.	Route ID	Total Travel Distance (meters)	Total Travel Time (min)	Use Elevators
P1	R1 R2	831.1 858.7	9.8 10.7	yes no
P2	R3 R4	801.7 829.3	9.5 10.3	yes no
P3	R5 R6	787.3 814.9	9.3 10.2	yes no
P4	R7 R8	875.4 905.2	10.5 11.2	yes no
P5	R9 R10	827.2 856.9	9.8 10.5	yes no

## Abbreviations

The following abbreviations are used in this manuscript:

VGI	Volunteered geographic information
OSM	Open Street Map
SLAM	Simultaneous localization and mapping
IMU	Inertial measurement unit
BIM	Building information
CityGML	City Geographic Markup Language
IFC	Industry Foundation Classes
OGC	Open Geospatial Consortium
F3DB	Full 3D buildings
UML	Unified Modeling Language
JOSM	Java OpenStreetMap
